# Long‐Term Observation of Orbital Development in Patients With Retinoblastoma Following Unilateral Enucleation

**DOI:** 10.1002/cam4.71282

**Published:** 2025-09-26

**Authors:** Nan Wang, Feng Ke, Jing Li, Tingting Ren, Rui Liu, Fuxiao Luan, Liangyuan Xu, Jianmin Ma

**Affiliations:** ^1^ Beijing Tongren Hospital, Capital Medical University Beijing China; ^2^ Shiyan Renmin Hospital Shiyan China; ^3^ Beijing Chaoyang Hospital, Capital Medical University Beijing China

**Keywords:** enucleation, long‐term observation, orbital development, orbital implants, retinoblastoma

## Abstract

**Background:**

This study aims to investigate bilateral orbital development differences and influencing factors in retinoblastoma patients undergoing unilateral enucleation.

**Methods:**

A retrospective comparative analysis was performed on patients from Beijing Tongren Hospital (January 2011–December 2020). Preoperative and 3 months, 1 year, 3 years postoperative, and the final follow‐up imaging data were collected, with bilateral orbital volumes reconstructed using ITK‐SNAP software.

**Results:**

Thirty‐nine patients were followed for an average of 7.7 ± 2.45 years. Average orbital volumes (mm^3^) for the surgical and nonsurgical sides before surgery were 14,323.81 ± 4596.60 and 14,457.93 ± 4732.26 (*p* = 0.330). Postoperative volumes at 3 months were 16,481.84 ± 4034.21 and 16,866.45 ± 3999.71 (*p* = 0.007). At 12 months, volumes were 16,798.16 ± 3323.33 and 18,119.16 ± 3840.27 (*p* = 0.000). At 36 months, volumes were 18,758.26 ± 2917.35 and 19,973.55 ± 3189.83 (*p* = 0.000). The last follow‐up volumes were 20,523.78 ± 3221.20 and 21,576.78 ± 3381.96 (*p* = 0.000). Bilateral volume differences were 2.28%, 7.29%, 6.08%, and 4.88% at 3 months, 12 months, 36 months after operation, and final follow‐up. The growth trajectory on the surgical side demonstrated growth restrictions, accompanied by a shift in the growth peak. Factors affecting development included the age at the time of operation and the type of orbital implants.

**Conclusions:**

Volumetric analysis revealed active orbital development between 3 and 12 months post‐enucleation, followed by a significant plateau phase. The final orbital volume deficit in the surgical orbit stabilized at approximately 5% compared to the non‐operated orbit. Hydrogel implants demonstrated a trade‐off: potentially reduced volume deficit but higher complication risks.

## 1 Introduction

With the advancement of retinoblastoma (RB) treatment methods, the survival rate of patients in high‐income countries can exceed 95%. In contrast, the overall global survival rate is < 30%. This disparity is mainly due to patients in low‐ and middle‐income countries often experiencing delayed medical treatment and late‐stage diagnoses. As a result, the rate of enucleation remains high. In China, for example, the enucleation rate can reach as high as 70% [1], making RB a leading cause of enucleation in children [2].

Orbital development relies on global stimulation and occurs relatively quickly in early life. By age 3, orbital development reaches approximately 80% of the adult level; by age 7, it is about 94% [3]. However, many children with RB are diagnosed before the age of 3, and enucleation is often performed early in life. The early loss of the globe can significantly affect the growth and development of the orbit, and clinical observations have noted restricted facial development following enucleation.

This study conducts long‐term follow‐ups of patients with unilateral enucleation due to RB and uses imaging reconstruction to calculate bilateral orbital volumes. The goal is to explore the differences in bilateral orbital development and identify the influencing factors.

## 2 Methods

We gathered data on children diagnosed with RB who were admitted to Beijing Tongren Hospital between January 1, 2011 and December 31, 2020. The inclusion criteria for this study comprised patients who underwent enucleation combined with primary orbital implantation, were treated and monitored according to a predefined plan, and had complete postoperative imaging data available. Exclusion criteria: (a) patients who had orbital implants removed due to orbital metastasis or cellulitis, (b) patients diagnosed with severe systemic diseases, (c) patients diagnosed with other malignant tumors and (d) patients who had previously received orbital or cranial radiotherapy (due to known significant inhibitory effects on bone growth). Implant selection was institutionally mandated: hydroxyapatite spheres were utilized except during 2015–2018 when hydrogel implants were exclusively available due to supply agreements. This non‐randomized but clinically neutral allocation enabled retrospective comparison of materials. Age stratification refers exclusively to the patient's age at enucleation surgery. Follow‐up intervals were calculated from the surgical date.

**FIGURE 1:**
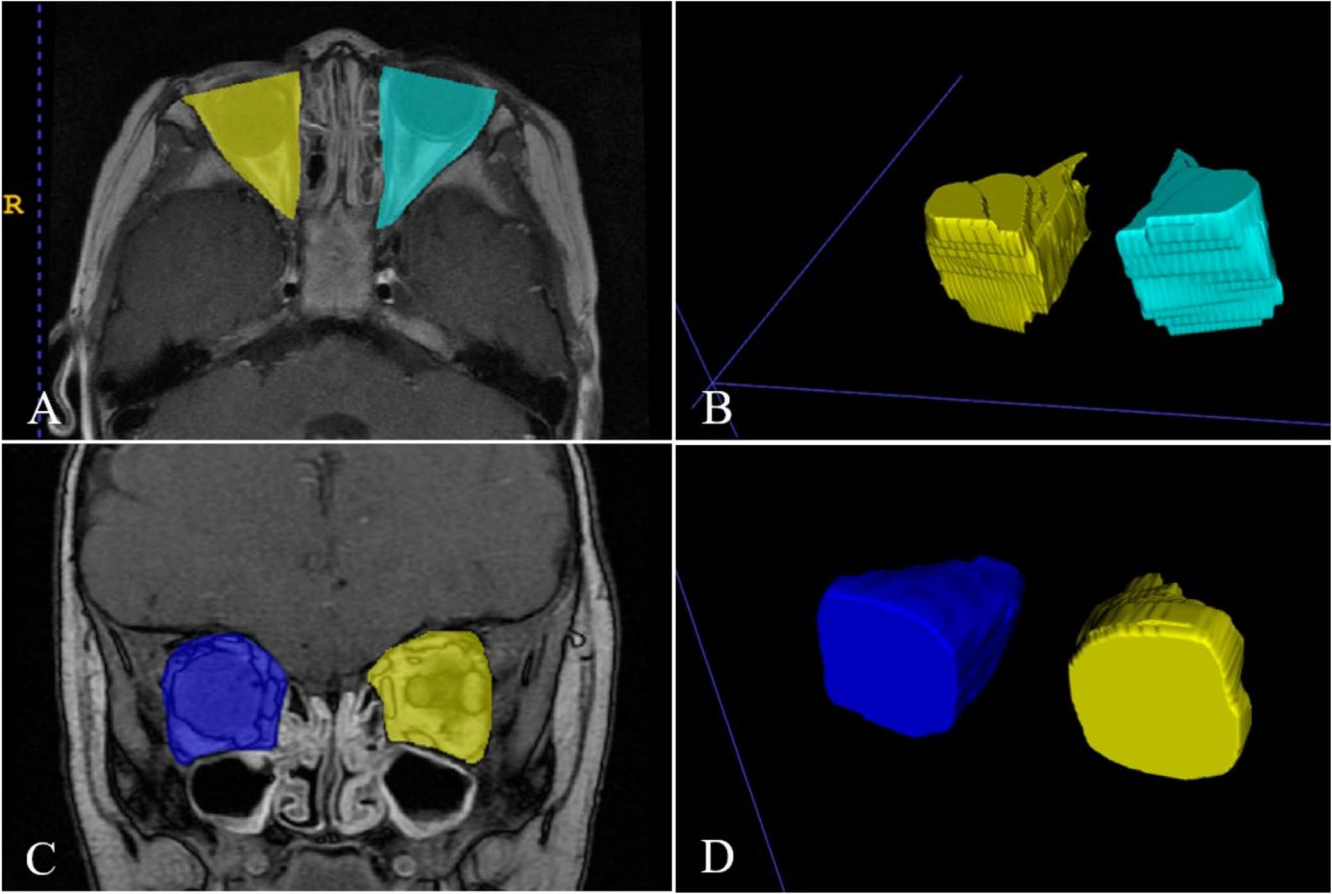
ITK‐SNAP orbital volume reconstruction. (A) Horizontal orbital margin delineation; (B) horizontal orbital volume reconstruction; (C) coronal orbital margin delineation; (D) coronal orbital volume reconstruction.

MRI was exclusively used for orbital volumetry to avoid ionizing radiation in this radiosensitive population. This aligns with recommendations for RB management. MRI images were collected at several time points: before the operation, 3 months post‐operation, 1 year post‐operation, 3 years post‐operation, and during the final follow‐up (conducted uniformly in August 2024 using standardized 3T MRI protocols with identical acquisition parameters across all participants). This surveillance interval was designed by synthesizing: (a) consensus recommendations for RB early‐phase monitoring, (b) evidence of first‐year complication clusters post‐enucleation from our cohort study [4], and (c) institutionally validated clinical protocols. We conducted orbital volumetry through systematic delineation on axial and coronal MRI sequences. The total orbital volume was measured on axial planes, initiating at the precise interface where hypointense bony signal (T1/T2) transitions to hyperintense orbital contents. Anterior boundaries were anatomically anchored at the anterior lacrimal crest nasally (marking the frontal/lacrimal/maxillary confluence) and the frontosphenoidal suture temporally, with tracing extending posteriorly to the optic canal convergence defining the orbital apex. Conversely, the posterior orbital volume was quantified on coronal sequences starting at the first slice posterior to the lateral canthal ligament (identified by its characteristic T2 hypointense fibrillar signature). Delineation followed the hypointense bony contour encircling hyperintense orbital soft tissues, terminating at the “orbital butterfly” configuration formed by the superior orbital fissure and optic canal confluence. The anterior orbital volume was derived computationally as the volumetric difference (anterior orbital volume = total orbital volume − posterior orbital volume), corresponding anatomically to the pre‐canthal space housing critical prosthetic interface structures. Orbital volume measurements were performed using ITK‐SNAP software (version 3.4.0, http://www.itksnap.org), a validated tool for medical image segmentation. After completing the outline, we reconstructed the orbital morphology and measured the volume using “Volumes and Statistics” in ITK‐SNAP (Figure 1). To minimize measurement error, the segmentation protocol involved manual delineation of orbital boundaries on MRI slices (slice thickness = 1 mm) by two independent observers (Author N.W. and Author F.K.), with anatomical landmarks as reference points as previously described, and the average of their results was taken. For datasets with discrepancies > 5%, a third reviewer (Author J.L.) conducted confirmation to ensure data accuracy. The difference between the horizontal orbital volume and the coronal orbital volume was identified as the anterior orbital volume. The study protocol was approved by the Ethics Committee of Beijing Tongren Hospital, affiliated with the Capital Medical University, and adhered to the tenets of the Declaration of Helsinki. The requirement for informed consent was waived owing to the retrospective design of the study.

All analyses were conducted using SPSS 20.0 (IBM Corp). Categorical variables are summarized as counts (percentages), with group differences evaluated by chi‐square test and Fisher's precision test. Continuous variables were assessed for normality using Shapiro–Wilk tests; normally distributed data were compared with independent *t*‐tests, while nonparametric data used Mann–Whitney *U* tests. Statistical significance was set at *p* < 0.05 (two‐tailed).

## 3 Results

### 3.1 Clinical Features

This study included 39 patients. Among the participants, 56.4% (22/39) were male, and 43.6% (17/39) were female. Binocular disease was documented in 23.1% (9/39) of the cases. Furthermore, 56.4% (22/39) affected the right eye, while 43.6% (17/39) were left. The disease stages were classified as follows: 35.9% (14/39) as stage D, 61.5% (24/39) as stage E, and 2.6% (1/39) as unknown. Geographically, 51.3% (20/39) of the patients resided in urban areas, while 48.7% (19/39) were from rural areas. The ages of the patients ranged from 2 to 124 months, with a median age of 9 (4, 26) months.

The lag period varied from 0 to 188 months, with a median of 5 (2, 27) months. The time from discovery to surgery ranged from 0 to 23 months, with a median of 2 (1, 4) months. High‐risk histopathological factors were noted in 28.9% (11/38).

Regarding the type of implants used, 23.1% (9/39) received hydrogel implants, while 76.9% (30/39) were fitted with hydroxyapatite (HA) implants. Enucleation was the initial treatment for 59.0% (23/39) of patients, while 41.0% (16/39) underwent chemotherapy before surgery. Following the operation, 66.7% (26/39) of patients received chemotherapy.

### 3.2 Postoperative Complications

The mean follow‐up duration from surgery to final evaluation was 92.4 ± 29.4 months (range: 48–144 months). Postoperative complications were noted in 17.95% of the patients (7/39), including one case of orbital cellulitis, two cases of implant exposure, two cases of conjunctival cysts, and five cases of ptosis. The rate of postoperative complications was significantly higher in patients from rural areas compared to those from urban regions (31.6% vs. 5.0%, *p* = 0.024). Furthermore, 37.5% of children under 7 months of age at the time of surgery experienced complications, which was markedly greater than the 4.3% seen in those aged 7 months or older (*p* = 0.007). Additionally, the hydrogel implantation group exhibited a higher incidence of postoperative complications compared to the HA implant group (33.3% vs. 13.8%, *p* = 0.186). However, factors such as gender, unilateral or bilateral diseases, and chemotherapy showed no significant effect on the occurrence of complications (Table 1).

**TABLE 1:** Association between clinical characteristics and postoperative complications.

	No complication group, *n* (%)	Complication group, *n* (%)	*p*
Gender			
Male	17 (77.3)	5 (22.7)	0.368
Female	15 (88.2)	2 (11.8)	
Disease laterality			
Bilateral	7 (77.8)	2 (22.2)	0.703
Unilateral	25 (83.3)	5 (16.7)	
Disease stage			
Stage D	12 (85.7)	2 (14.3)	0.610
Stage E	19 (79.2)	5 (20.8)	
Orbital implant			
Hydrogel	6 (66.7)	3 (33.3)	0.186
HA	25 (86.2)	4 (13.8)	
Region			
Rural areas	13 (68.4)	6 (31.6)	**0.024**
Urban areas	19 (95)	1 (5)	
Preoperative chemotherapy			
Yes	13 (81.3)	3 (18.8)	0.914
No	19 (82.6)	4 (17.4)	
≤ 6 months			
Yes	10 (62.5)	6 (37.5)	**0.007**
No	22 (95.7)	1 (4.3)	

*Note:* Comparisons between subgroups were analyzed by chi‐square/Fisher's exact test. The bold values indicate statistical significance, defined as a *p*‐value of less than 0.05.

### 3.3 Orbital Volume

**FIGURE 2:**
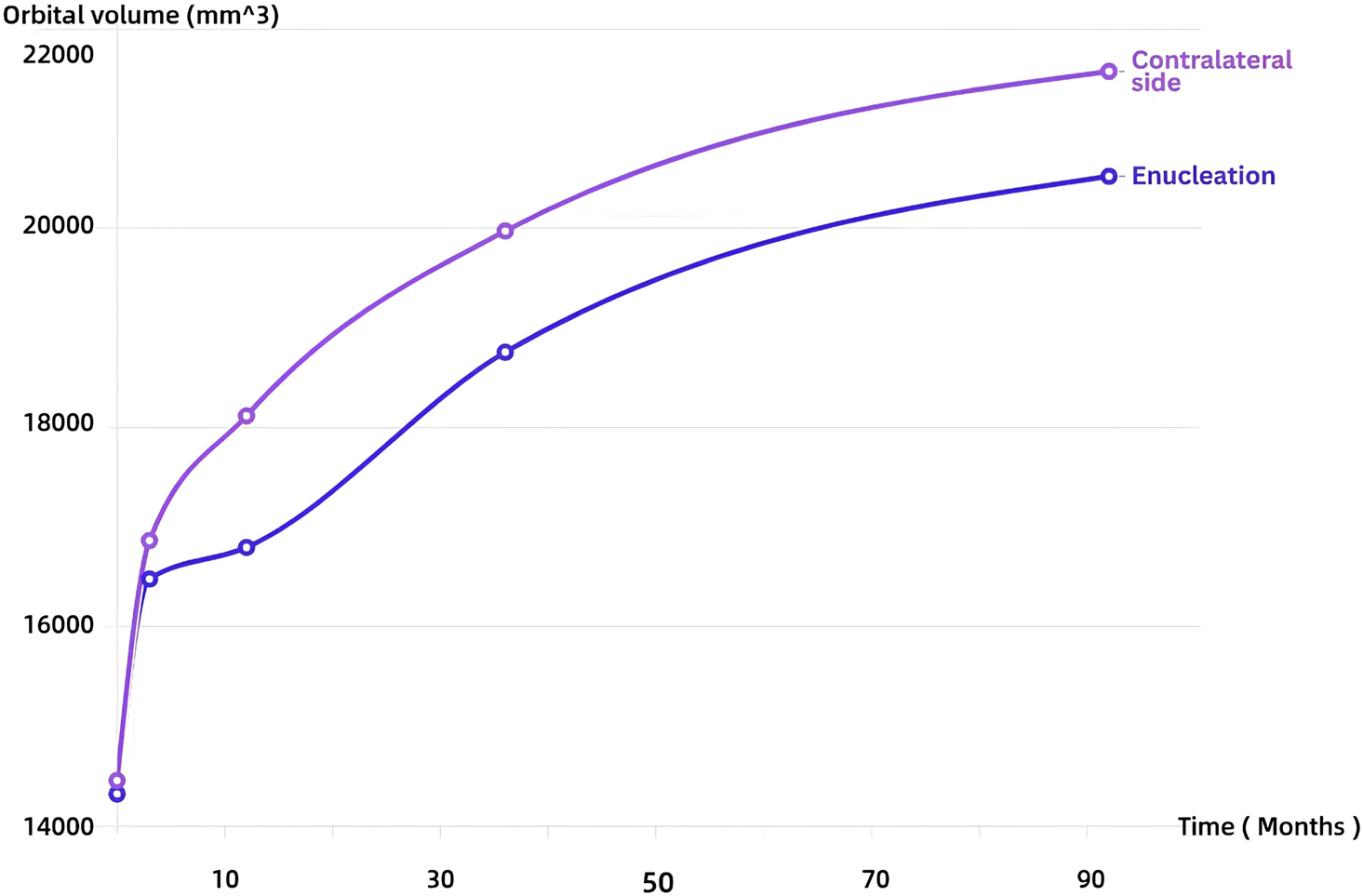
Curve of orbital volume after unilateral enucleation.

**FIGURE 3:**
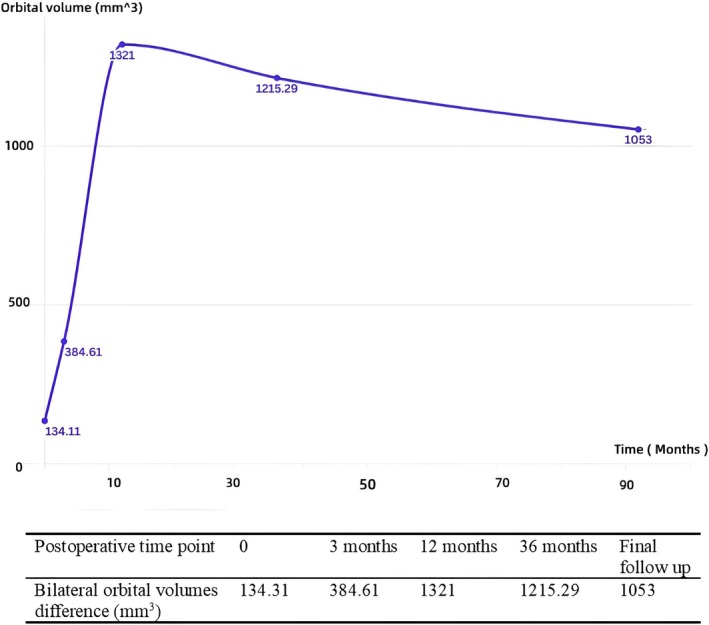
Curve of bilateral orbital volume difference after unilateral enucleation.

Longitudinal analysis revealed asynchronous orbital development following unilateral enucleation, with surgically treated orbits exhibiting significantly impaired growth during the critical 3‐ to 12‐month postoperative period. While preoperative mean orbital volumes showed no statistically significant difference between sides (*p* = 0.330), significant disparities emerged at all postoperative intervals: 3 months (*p* = 0.007), 12 months (*p* = 0.000), 36 months (*p* = 0.000), and final follow‐up (*p* = 0.001) as documented in Table 2. The volumetric growth curve demonstrated restricted expansion on the surgical side with delayed peak growth relative to physiological expectations (Figure 2). Bilateral volume differences reached maximal asymmetry at 12 months postoperatively (7.29% disparity) before subsequently declining (Figure 3).

**TABLE 2:** Orbital volume of unilateral enucleation RB patients.

	Orbital volume (mm^3^)		*p*
	Enucleation sides	Nonsurgical sides	
Preoperative	14,323.81 ± 4596.60	14,457.93 ± 4732.26	0.330
3 months post‐operation	16,481.84 ± 4034.21	16,866.45 ± 3999.71	**0.007**
12 months post‐operation	16,798.16 ± 3323.33	18,119.16 ± 3840.27	**0.000**
36 months post‐operation	18,758.26 ± 2917.35	19,973.55 ± 3189.83	**0.000**
Last follow‐up	20,523.78 ± 3221.20	21,576.78 ± 3381.96	**0.000**

*Note:* Data are expressed as mean orbital volume (±SD) between operated and fellow orbits at specific time points using *t*‐tests. The bold values indicate statistical significance, defined as a *p*‐value of less than 0.05.

Calculation of growth rates using the formula ΔRate = [(current volume − previous volume)/previous volume] × 100% revealed statistically significant differential growth during the 3‐ (*p* = 0.015) and 12‐month (*p* = 0.015) interval, where surgical orbits lagged substantially behind contralateral sides (Table 3). Subgroup analysis of peak 12‐month asymmetry identified three significant modifiers: enucleation before age 7 months (8.00% ± 4.11% vs. 4.61% ± 1.39%, *p* = 0.047), hydrogel implant usage (4.08% ± 1.70% vs. 7.26% ± 3.75%, *p* = 0.025), and rural residence (6.08% ± 3.11% vs. 7.65% ± 4.29%, *p* = 0.025), all demonstrating significant associations with orbital development as presented in Table 4.

**TABLE 3:** Growth rate differences (ΔRate) between surgical and contralateral orbits.

	ΔRate (%)		*p*
	Enucleation sides	Nonsurgical sides	
3 months post‐operation	18.33% ± 4.70%	21.4% ± 5.15%	**0.015**
12 months post‐operation	5.12% ± 2.20%	8.89% ± 7.95%	**0.015**
36 months post‐operation	10.43% ± 3.09%	8.04% ± 3.48%	0.182
Last follow‐up	13.71% ± 5.43%	11.94% ± 5.36%	0.242

*Note:* ΔRate = [(current volume − previous volume)/previous volume] × 100%. Data are expressed as mean ΔRate (±SD) between operated and fellow orbits at specific time points, using *t*‐tests. The bold values indicate statistical significance, defined as a *p*‐value of less than 0.05.

**TABLE 4:** Factors associated with orbital development after unilateral enucleation.

	Percentage reduction of orbital volume on surgical side		*p*
	Yes (*n*)	No (*n*)	
Age of operation ≤ 6 months	8.00% ± 4.11% (16)	4.61% ± 1.39% (23)	**0.047**
Rural areas	6.08% ± 3.11% (19)	7.65% ± 4.29% (20)	0.342
Bilateral diseases	5.74% ± 1.79% (9)	7.31% ± 4.19% (30)	0.429
Postoperative chemotherapy	7.22% ± 3.49% (26)	5.75% ± 5.36% (13)	0.495
Stage D	5.90% ± 4.11% (14)	7.34% ± 3.26% (25)	0.371
Secondary glaucoma	5.90% ± 3.94% (13)	7.70% ± 3.87% (24)	0.298
Hydrogel implant	4.08% ± 1.70% (9)	7.26% ± 3.75% (30)	**0.025**
HHFs	7.43% ± 3.89% (11)	5.77% ± 4.04% (27)	0.706
Optic nerve invasion	8.72% ± 4.09% (12)	6.20% ± 3.56% (26)	0.150
Massive choroidal invasion	7.00% ± 3.91% (7)	6.61% ± 3.89% (31)	0.911
Age of operation ≤ 12 months	7.87% ± 3.89% (24)	6.14% ± 3.73% (15)	0.290

*Note:* The comparison of mean orbital volume (±SD) between different subgroups using *t*‐tests. The bold values indicate statistical significance, defined as a *p*‐value of less than 0.05.

### 3.4 Posterior Orbital Volume

The average preoperative posterior orbital volumes for the surgical and nonsurgical sides were 12,883.72 ± 4095.08 and 12,905.00 ± 3938.16 (*p* = 0.823). Three months after the operation, the values were 14,740.84 ± 3448.64 and 15,176.49 ± 3757.93 (*p* = 0.151) for the surgical and nonsurgical sides. At 12 months, the figures were 15,474.20 ± 2997.83 and 16,253.33 ± 2897.77 (*p* = 0.002). While at 36 months, they were 16,918.65 ± 3877.29 and 17,928.69 ± 4102.77 (*p* = 0.017). The final follow‐up recorded values of 18,005.09 ± 2941.59 and 18,550.12 ± 3077.28 (*p* = 0.048) respectively (Table 5). The growth curve for the posterior orbital volume illustrated that the growth on the surgical side was restricted, with the peak of growth occurring later than expected. The difference between the nonsurgical and surgical sides was calculated at each time point, showing that this difference peaked at 36 months before decreasing slightly thereafter.

**TABLE 5:** Posterior orbital volume of unilateral enucleation RB patients.

	Posterior orbital volume (mm^3^)		*p*
	Enucleation sides	Nonsurgical sides	
Preoperative	12,883.72 ± 4095.08	12,905.00 ± 3938.16	0.823
3 months post‐operation	14,740.84 ± 3448.64	15,176.49 ± 3757.93	0.151
12 months post‐operation	15,474.20 ± 2997.83	16,253.33 ± 2897.77	**0.002**
36 months post‐operation	16,918.65 ± 3877.29	17,928.69 ± 4102.77	**0.017**
Last follow‐up	18,005.09 ± 2941.59	18,550.12 ± 3077.28	**0.048**

*Note:* Data are expressed as mean posterior orbital volume (±SD) between operated and fellow orbits at specific time points using *t*‐tests. The bold values indicate statistical significance, defined as a *p*‐value of less than 0.05.

### 3.5 Anterior Orbital Volume

Before surgery, the average anterior orbital volumes were 821.71 (426.35, 2365.15) for the surgical side and 1106.60 (199.40, 3079.51) for the nonsurgical side (*p* = 0.797). Three months post‐surgery, the volumes recorded were 1550.54 (353.08, 3999.85) for the surgical side and 2020.05 (734.58, 2872.13) for the nonsurgical side (*p* = 0.808). At the 12‐month postoperative mark, the values were 1540.90 (678.88, 2610.23) for the surgical side and 1828.00 (732.30, 3279.95) for the nonsurgical side (*p* = 0.158). By the 36‐month follow‐up, the surgical side recorded a volume of 1564.30 (150.88, 3470.40) and 1964.10 (677.95, 5329.20) for the nonsurgical side (*p* = 0.551). At the last follow‐up, they were 3168.90 (1566.10, 4698.50) and 3763.50 (1180.90, 4781.60), respectively (*p* = 0.091) (Table 6), indicating that the posterior orbital volume mainly caused the difference in bilateral orbital volumes.

**TABLE 6:** Anterior orbital volume of unilateral enucleation RB patients.

	Anterior orbital volume (mm^3^)		*p*
	Enucleation sides	Nonsurgical sides	
Preoperative	821.71 (426.35, 2365.15)	1106.60 (199.40, 3079.51)	0.797
3 months post‐operation	1550.54 (353.08, 3999.85)	2020.05 (734.58, 2872.13)	0.808
12 months post‐operation	1540.90 (678.88, 2610.23)	1828.00 (732.30, 3279.95)	0.158
36 months post‐operation	1564.30 (150.88, 3470.40)	1964.10 (677.95, 5329.20)	0.551
Last follow‐up	3168.90 (1566.10, 4698.50)	3763.50 (1180.90, 4781.60)	0.091

*Note:* Data are expressed as median (IQR) between operated and fellow orbits at specific time points using nonparametric tests.

### 3.6 Orbital Volume Grouped by Age

We conducted age‐stratified analyses by grouping patients according to their age at the time of each MRI examination. Orbital volumes (mm^3^) for surgical versus nonsurgical sides across these age‐stratified cohorts, analyzed using paired‐sample *t*‐tests, are presented in Table 7:

– At the age of 1: Surgical side 14,220.05 ± 1468.77, nonsurgical side 14,612.53 ± 1463.14, *p* = 0.005.
– At the age of 2: Surgical side 16,756.48 ± 1906.51, nonsurgical side 17,705.56 ± 1661.78, *p* = 0.001.
– At the age of 3: Surgical side 17,989.61 ± 1502.21, nonsurgical side 19,095.49 ± 1654.41, *p* = 0.000.
– At the age of 4: Surgical side 17,785.93 ± 2042.26, nonsurgical side 18,818.97 ± 2273.02, *p* = 0.000.
– At the age of 5: Surgical side 18,056.00 ± 1426.32, nonsurgical side 19,390.38 ± 2085.65, *p* = 0.010.
– At the age of 6: Surgical side 19,871.30 ± 2865.53, nonsurgical side 20,968.38 ± 2601.00, *p* = 0.003.

**TABLE 7:** Orbital volume grouped by age of unilateral enucleation RB patients.

	Orbital volume (mm^3^)		*p*
	Enucleation sides	Nonsurgical sides	
The age of 1	14,220.05 ± 1468.77	14,612.53 ± 1463.14	0.005
The age of 2	16,756.48 ± 1906.51	17,705.56 ± 1661.78	0.001
The age of 3	17,989.61 ± 1502.21	19,095.49 ± 1654.41	0.000
The age of 4	17,785.93 ± 2042.26	18,818.97 ± 2273.02	0.000
The age of 5	18,056.00 ± 1426.32	19,390.38 ± 2085.65	0.010
The age of 6	19,871.30 ± 2865.53	20,968.38 ± 2601.00	0.003

*Note:* The presentation of mean orbital volume (±SD) stratified by age and the use of *t*‐tests for comparisons.

**FIGURE 4:**
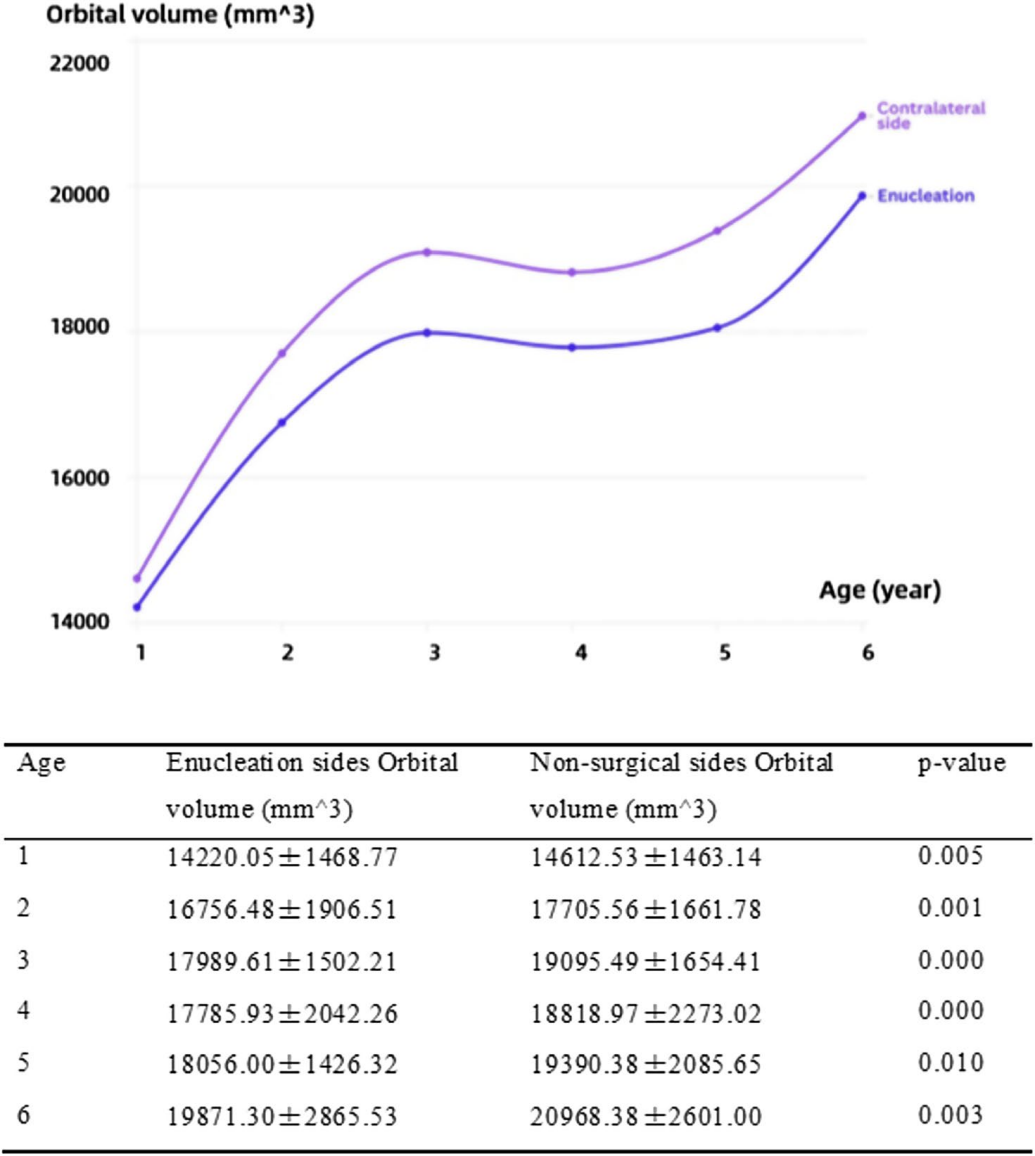
Growth curve of bilateral orbital volumes.

The growth curve of orbital volume on the surgical side demonstrated a rightward‐lagging growth pattern compared to the nonsurgical side, indicating that its growth and development were delayed and restricted (Figure 4).

## 4 Discussion

Despite various eye protection treatments, over half of patients in China still require enucleation [1]. Pediatric patients face more severe complications, including skeletal retardation and facial asymmetry [5]. Orbital implants are used to compensate for volume loss and stimulate bone development. However, the eye continues to grow after age 2, limiting the effectiveness of fixed‐size implants over time [6].

Restricted orbital development has multifaceted clinical consequences. Esthetically, it often leads to facial asymmetry, particularly in the midfacial region, due to impaired orbital growth [5]. Functionally, inadequate orbital volume and bony structure can result in difficulties in prosthetic eye fitting and retention, with long‐term complications including conjunctival fornix contracture and implant exposure [2]. Furthermore, growing evidence indicates that such orbital deformities may contribute to psychosocial challenges in pediatric patients, including reduced self‐esteem, social anxiety, and long‐term emotional distress, which can persist into adulthood [7]. These combined esthetic, functional, and psychological impacts underscore the importance of early intervention strategies to mitigate orbital developmental restrictions following enucleation.

Although CT remains the gold standard for delineating bony anatomy, most of RB patients harbor RB1 mutations, conferring a lifelong elevated risk of secondary malignancies. Current guidelines strongly recommend minimizing radiation exposure in this population. Our MRI‐based protocol prioritized patient safety given the established radiosensitivity in RB. MRI was exclusively employed for orbital volumetry to avoid ionizing radiation in these at‐risk individuals [8]. Importantly, our exclusion of patients with radiotherapy history mitigates potential confounding effects on orbital development. Bilateral differences in orbital volume were noted 3 months post‐operation, with a 2.2% reduction on the surgical side compared to the nonsurgical side. Over time, this discrepancy initially increased and then decreased, peaking at 7.29% 12 months after the procedure. Subsequently, the difference gradually declined and stabilized at 4.88%, suggesting that the development of the surgical side's orbit not only lagged in terms of growth but also regressed temporally, demonstrating a growth curve that shifts downward and to the right. This finding stands in contrast to the prior research by Kruglov et al. [5], who reported no significant orbital volume asymmetry in patients with unilateral retinoblastoma who underwent enucleation and primary HA implantation, as evaluated via MRI examinations conducted 5 years post‐enucleation. This discrepancy is likely attributable to the differing timing of assessments: whereas their study focused on long‐term outcomes at the 5‐year mark, our analysis emphasizes the early postoperative period, which may capture more pronounced developmental changes that diminish or stabilize over extended follow‐up. Notably, the peak difference in bilateral orbital volumes occurred 12 months after operation, indicating that this timeframe is critical for mitigating the development of the affected orbit. Notably, the peak bilateral orbital volume discrepancy occurring at 12 months postoperatively underscores the critical importance of this period for mitigating developmental impairment in the affected orbit. This phenomenon likely stems from three coexisting biomechanical deficits during early implant integration: (1) delayed force transduction capacity until complete fibrovascularization, (2) irreversible loss of physiological vitreous pressure dynamics, and (3) nonphysiological tension vectors from reattached extraocular muscles. These deficits exert maximal impact during rapid growth phases where mechanical loading crucially regulates osteogenesis. Beyond this peak, progressive stabilization emerges through dual compensatory pathways: implant maturation restoring mechanotransduction function and developmental adaptation featuring contralateral growth deceleration. During this period, it is essential to reduce follow‐up intervals, increase follow‐up frequency, reinforce comparisons between the bilateral orbits, and replace the ocular prostheses film to minimize any adverse effects on orbital bone development. Early intervention and repair can significantly decrease facial asymmetry [9]. Our data cannot definitively resolve the implant timing controversy due to protocol‐mandated primary implantation. However, our age‐stratified data reveal a clinically meaningful trend: infants implanted ≤ 6 months exhibited nearly double the volume deficit of later‐implanted peers (8.00% vs. 4.61%). This aligns with biomechanical models suggesting reduced orbital expansion force during peak growth phases.

In this study, we observed that patients who underwent surgical procedures < 6 months prior showed more significant orbital volume asymmetry related to using smaller orbital implants. Notably, our previous work aligns with the current observations, as we documented bilateral orbital asymmetry in terms of external appearance, size, and orbital socket depression among patients treated during early infancy [10]. This consistency reinforces the notion that the immature orbital skeleton during the infantile period exhibits heightened vulnerability to developmental perturbations following surgical intervention. In this study, the anterior and posterior orbital volumes were measured separately for the first time, and discovered that the asymmetry primarily correlated with the posterior orbit. Analysis revealed characteristics indicative of growth restriction in the posterior orbit, with the peak growth phase being delayed; conversely, no significant variations were noted in the anterior orbital volume across different time points. When stratifying the subjects by age, we found that orbital volume in children escalated rapidly until the age of three, after which the disparity in bilateral orbital volumes stabilized. Notably, the enucleated orbit exhibited a rightward‐lagging growth trajectory in volume development compared with the contralateral side, suggesting temporal delay and spatial constraints in orbital maturation.

Despite the critical nature of selecting implant materials, there remains an absence of a universal standard worldwide [11]. An ideal implant would possess several essential qualities: excellent biocompatibility, the capacity to foster bone development and soft tissue growth, ease of implantation and fixation, superior esthetic and functional outcomes, and a reduced incidence of postoperative complications [12]. Within our investigation, we primarily used two types of orbital implants: the nonporous implants, represented by the self‐expanding hydrogel implants, and the porous implants, represented by HA implants. The porous implant facilitates fibrovascular ingrowth and vascularization, contributing to enhanced stability and safety while mitigating the risk of infection [12, 13, 14]. In contrast, with their self‐expanding properties, the hydrogel implants may promote the development of orbital bone and soft tissue. Our observations suggest that the hydrogel implants demonstrated superior efficacy in minimizing orbital volume asymmetry, although it is important to acknowledge the potential for increased risk of various postoperative complications. It is crucial to contextualize these findings within the extensive literature on intraorbital implant materials. While the current study highlights age‐ and implant material‐related volumetric changes, our research has identified both positive and negative bidirectional effects of hydrogel implants. However, other studies have demonstrated that nonporous implants do not pose a higher risk of complications compared to porous ones [15]. This distinction underscores the multifaceted nature of orbital reconstruction, where both developmental outcomes and complication rates must be taken into account in clinical decision‐making. Nevertheless, within our cohort, long‐term observations indicated that the complications encountered were primarily mild, and patients exhibited favorable prognoses posttreatment. Consequently, for experienced practitioners, hydrogel implants may be suitable for orbital implantation. However, it is noteworthy that prior studies have documented challenges associated with hydrogel implants in clinical settings over a decade, including uncontrolled expansion and degradation issues, which may compromise the implant's structural integrity and complicate eventual removal [16]. Moreover, hydrogel implants lack the capacity for vascularization and fixation within the orbit, thus presenting an opportunity for novel treatment approaches. Based on the observed orbital developmental delay on the surgical side, we tentatively suggest that early implantation of hydrogel implants may increase the opportunities for orbital bone development, with subsequent replacement by more stable HA implants to maintain long‐term orbital volume. However, it should be noted that this sequential strategy currently lacks direct clinical evidence to support its efficacy. This hypothesis still requires further clinical validation.

Several limitations of the present study should be acknowledged. While the 1.73% greater discrepancy in < 1‐year patients (7.87% ± 3.89 vs. 6.14% ± 3.73) did not reach statistical significance (*p* = 0.290), this clinically relevant trend demonstrates age‐dependent vulnerability consistent with our ≤ 6‐month subgroup findings—potentially reflecting sample size constraints in older cohorts. Implant type allocation was non‐randomized and supply‐dependent. Although this created comparable cohorts without clinical selection bias (hydrogel *n* = 9 vs. hydroxyapatite *n* = 30), the small hydrogel subgroup limits statistical power for material comparisons. Future studies should prospectively evaluate material impacts.

The abnormal orbit development in patients with RB following enucleation is a notable concern. The difference in bilateral orbital development peaked 12 months after the surgery and stabilized at approximately 5%. The period between 3 and 12 months post‐enucleation represents a critical window for mitigating volumetric restrictions in orbital development, underscoring the necessity of optimized follow‐up evaluations during this phase. Infants undergoing surgery at or below 6 months of age exhibited more pronounced orbital growth restriction compared to older peers. Long‐term orbital development outcomes for patients undergoing enucleation at or below 6 months of age remain unknown and warrant future investigation. While hydrogel implants showed less volume deficit (4.08% ± 1.70% vs. 7.26% ± 3.75% in hydroxyapatite), this subgroup (*n* = 9) had significantly higher complication rates (33.3% vs. 13.8%). Given limited power and retrospective design, these observations require validation in prospective trials before clinical application.

## Author Contributions

**Nan Wang:** data curation (lead), methodology (equal), validation (equal), writing – original draft (equal). **Feng Ke:** conceptualization (equal), supervision (equal), validation (equal), writing – original draft (equal). **Jing Li:** methodology (equal), visualization (equal). **Tingting Ren:** data curation (equal), visualization (equal). **Rui Liu:** project administration (equal), visualization (equal). **Fuxiao Luan:** conceptualization (equal), validation (equal). **Liangyuan Xu:** data curation (equal), investigation (equal). **Jianmin Ma:** funding acquisition (equal), resources (equal), supervision (equal), writing – review and editing (lead).

## Ethics Statement

The study protocol was approved by the Ethics Committee of Beijing Tongren Hospital, affiliated with the Capital Medical University, and adhered to the tenets of the Declaration of Helsinki. The requirement for informed consent was waived owing to the retrospective design of the study.

## Conflicts of Interest

The authors declare no conflicts of interest.

## Data Availability

The data that support the findings of this study are available from the corresponding author upon reasonable request.
